# A Pilot Study on BMI, Serum Testosterone and Estradiol Levels in Allergic Male Patients

**DOI:** 10.3889/oamjms.2015.101

**Published:** 2015-09-22

**Authors:** Violeta Lokaj-Berisha, Besa Gacaferri-Lumezi, Naser Berisha, Sanije Gashi-Hoxha

**Affiliations:** 1 *Department of Physiology & Immunology, Faculty of Medicine, University of Prishtina, Republic of Kosovo*; 2 *Departments of OB & GYNE, University Clinical Centre, Prishtina, Republic of Kosovo*; 3 *National Institute of Public Health, Prishtina, Republic of Kosovo*

**Keywords:** allergy, BMI, estradiol, male, testosterone

## Abstract

**BACKGROUND:**

The dramatic increase in the prevalence of high body mass index (BMI) increases the prevalence of allergic diseases, both in adults and children and obesity is associated with hypogonadism in adult males.

**AIM:**

We aimed to evaluate the effect of high body mass index on plasma concentrations of testosterone and estradiol in young pubertal and adult males with allergic diseases.

**MATERIAL AND METHODS:**

Morning fasting blood samples were obtained form 51 allergic patients and 6 healthy volunteer males between the ages 11-57 years (Mean 26.9, DS ± 11.9 years). Total testosterone, estradiol, FSH and LH concentrations were measured by radioimmunoassay. All participants were subjected to skin prick tests with test kit G aeroallergens, and BMI was calculated according to the body weight divided by the square of height (kg/m^2^).

**RESULTS:**

Low levels of testosterone and high levels of estradiol were associated with high BMI only in patients with asthma/rhinitis, but not in asthma patients. Allergic dermatitis/urticaria group along with healthy controls were overweight but within normal ranges for total testosterone and estradiol concentrations. Patients with allergic rhinitis were within normal ranges for BMI, total testosterone and estradiol concentrations.

**CONCLUSION:**

High BMI is not always associated with low levels of testosterone and high levels of estradiol in our patients with allergic diseases, but low levels of testosterone are present in patients with asthma and asthma/rhinitis although not among patients with rhinitis only. Our results should be confirmed in a larger group of participants.

## Introduction

The dramatic increase in the prevalence of obesity increases the prevalence of allergic diseases, especially IgE -mediated atopic ones, both in adults and children [1-3]. This phenomenon, in recent decades, has challenged scientists worldwide to review the possible causes and consequences of these diseases, including sex hormones, although the accompanying mechanisms are not clear [4, 5]. Gender differences, age of onset, BMI, social and environmental problems have been the subject of several epidemiological and clinical studies [4-6]. Some data from animal studies suggest that estrogen and testosterone can regulate specific and non-specific immune responses, revealing immune-modulating effects of sex hormones [7-9]. Further, testosterone acts as immune-suppressant and estrogen as pro-inflammatory, accordingly the first one is likely to be protective for men, but the latter is implicated in allergies [10].

Men, as teenagers, have high levels of testosterone and low levels of estrogen, but getting older, testosterone levels decline and estrogen levels increase, so low levels of testosterone usually correspond with higher levels of estrogen. However, despite the fact that testosterone levels decline with increasing age of men, it can often be associated with several other adverse conditions, such as obesity or diabetes [11]. Conversion of testosterone to estrogen is due to aromatase reaction, more abundant in fat cells of adipose tissue, so the more fat the human body has the more aromatase and consequently the more estrogen [12]. Given the fact that obesity is associated with allergic diseases, one may speculate that low levels of testosterone and high levels of estradiol in atopic obese men, regardless of any age may be associated with allergic diseases [13].

Based on conflicting statements reported in clinical and epidemiological studies, about the role of testosterone and estradiol in allergy, our goal was to elucidate any relationship between sex hormones and allergic diseases related to increased body mass index (BMI) and age of our male patients [14, 15].

## Material and Methods

### Subjects

Our study enrolled fifty seven (57) male subjects: 51 were patients who experienced allergy symptoms of asthma (A), asthma/rhinitis (A/Rh), rhinitis (Rh) and allergic dermatitis/urticaria (derm/urtic) and six (6) subjects from medical staff were healthy volunteers. Participants of the study group were consecutive patients, referred to the Allergy & Clinical Immunology outpatient service at the UCC Prishtina.

After being evaluated about their allergy symptoms and diagnosed with any of the above respiratory or skin diseases, they were recruited in voluntary bases. Informed written consent was obtained from all participants who were then included in the research. To each subject was handed over a study questionnaire requesting demographic data, family history of atopy, respiratory symptoms and smoking habits as well as not to be on prescription medication believed to affect hormone levels. They were measured for height and weight, using stadiometer and electronic scale. The BMI was calculated according to the body weight divided by the square of height (kg/m^2^) and was classified as low (BMI, <18.5 kg/m^2^), normal (18.5-24.9 kg/m^2^), overweight (25-29.9 kg/m^2^) and obese (BMI, > or = 30 kg/m^2^), according to the World Health Organization (WHO), the US Preventive Services Task Force and the International Obesity Task Force.

Patients, based on their diagnoses, were divided in four groups: the rhinitis group, the asthma/rhinitis group, asthma without rhinitis and allergic dermatitis/urticaria group. The controls were healthy volunteers selected according to the answers about not having, nor ever had, symptoms of respiratory allergies (coughing, sneezing, itching and nose discharge); absence of self-reported chronic disease (diabetes, heart disease, high blood pressure, cancer, and ulcer) and not on prescription medication believed to affect hormone levels. The average age of men included in the survey was 26.9 years (SD ± 11.9 years), range 11-57 years. The protocol for the research project has been approved by the Medical Faculty ethics committee.

### Blood sampling

For the measurement of serum concentration of total testosterone, estradiol, follicle stimulating hormone (FSH) and luteinizing hormone (LH), venous blood (2 mL) was obtained between 8:00 and 10:00 a.m. Blood samples were allowed to clot at room temperature and the serum was separated by centrifugation at 1,200×g for 10 minutes and stored at -80°C. Sera were thawed at room temperature before measurements. Total testosterone, estradiol, FSH and LH levels were measured by RIA, using Immunotech Beckman Coulter company products (France) at the laboratory of Endocrinology, Institute of Physiology, UCC Prishtina. Bound radioactivity was calculated with Gamma counter and expected normal ranges, according to manufacturer’s suggestion, for total testosterone were 9.0-41.6 nmol/L, estradiol 55-260 pmol/L, FSH 1.3 - 11.5 IU/L and LH 0.5-10.0 IU/L

### Skin prick test

All participants were subjected to SPT with test kit G aeroallergens, Allergopharma product (Reinbeck Germany), along with positive control – histamine (1 mg/ml) and negative – saline. Allergenic extracts included 3 groups of pollen: grasses (grasses/cereals, grasses, rye), trees (alder, hazel, birch, beech) and weeds (mugwort, E. plantain). The allergenic extract was placed on to the volar surface of forearm and introduced into the epidermis with sterile lancet (1 mm depth), new for each allergen. For each subject, 15 minutes later, both diameters of skin reaction were recorded and SPT was considered positive if diameter >3 mm. The patients were all allergic to at least one allergen extract, and some volunteers from the control group reacted positive too.

### Statistical analyses

Data are presented as the median and mean. Kruskal−Wallis variance analysis was used for screening differences among the five groups. Fisher exact test and Mann-Whitney test were used to compare the differences between two groups among healthy controls (positive or negative to skin prick test). *P*−values less than 0.05 were considered significant.

## Results

After assessing the age of the participants in this study, we found that the average age was 26.9 years (SD ± 11.9 years), but when we calculated the average age of the patients in groups with particular disease and healthy participants in the control group, we found that the A group consisted of younger subjects compared with other groups, especially those with Derm/Urtic which was characterized by the significantly higher average age value (KW = 9.61, P=0.04, P<0.05) (Table 1).

**Table 1:** Age, BMI and serum concentrations of total testosterone and estradiol in allergic patients and controls

	A (n=4)	A/Rh (n=10)	Rh (n=34)	Derm/urtic (n=3)	Controls (n=6)	P value
Age (Mean, SD)	14.8 (2.8)	28.2 (15.1)	26.1 (10.6)	34.0 (11.8)	33.5 (13.2)	0.047

BMI (Median, range)	22.4 (16.9-46)	25 (15.3-29.4)	23.4 (10.0-31.5)	28.4 (24.3-31.9)	25 (23.4-29.4)	0.290
N (%)						
Normal	3 (75.0)	5 (50.0)	20 (58.8)	1 (33.3)	2 (33.3)	
Overweight	-	5 (50.0)	13 (38.2)	1 (33.3)	4 (66.7)	
Obese	1 (25.0)	-	1 (2.9)	1 (33.3)	-	

Total Testosterone Nmol/L	1.6	9.8	17.2	17.9	16.3	0.115
(Median, range)	(1.3-15.1)	(0.2-35.1)	(0.7-40.0)	(11.03-22.3)	(2.6-26.3)
N (%)						
< 9.0	2 (50.0)	5 (50.0)	2 (5.9)	-	1 (16.7)	
9.0-41.6	2 (50.0)	5 (50.0)	32 (94.1)	3 (100.0)	5 (83.3)

Estradiol Pmol/L (Median, range)	188.2 (55-214)	178.7 (54-339)	160.8 (66-410)	177.2 (172-212)	179.5 (179-278)	0.996
N (%)						
< 55	1 (25.0)	1 (10.0)	-	-	1 (16.7)	
55-260	3 (75.0)	6 (60.0)	22 (64.7)	3 (100.0)	4 (66.7)	
> 260	3 (30.0)	12 (35.3)	-	1 (16.7)		

n - number of subjects; Rh – allergic rhinitis; A/Rh –allergic asthma with rhinitis; Derm/urtic – allergic dermatitis/acute urticaria; BMI- body mass index (normal= 18.5-24.9 kg/m^2^, overweight = 25-29.9 kg/m^2^, obese >30 kg/m^2^).

In addition, when we calculated the values for BMI, we found that 46% of all subjects had values above the normal range, including a high percentage of healthy controls (Table 1). However, average values for BMI between study subjects indicate that patients with A/Rh and Derm/Urtic along with healthy participants of control group were overweight (Table 1). In addition, the testosterone level was low in patients with A/Rh and much lower in the group with asthma, compared with other disease groups, as well as the healthy control group, but no significant differences between groups were recorded.

Also, estradiol concentration was above the normal level in a good percentage of the patients with A/Rh, Rh and healthy controls, but within the normal range in all patients with Derm/Urtic. However Median estradiol levels in all study groups were within the normal range, with no significant differences between groups (Table 1). Eventually, when assessing the concentration of FSH and LH, we found that the median value for both these hormones was the lowest in the group with A and the highest in the group with the Derm/Urtic, but no significant differences between groups (Table 2). Given the implication of BMI and age at the level of testosterone and estradiol, we context the correlation between age, BMI and variables such as testosterone and estradiol, in both groups of participants, patients regardless of the type of allergic disease and control group as well.

**Table 2:** Serum concentrations of FSH and LH in allergic patients and controls

Hormone levels	A (n=4)	A/Rh (n=10)	Rh (n=34)	Derm/urtic (n=3)	Controls (n=6)	P value
FSH IU/L (Median, range)	2.1 (1.4-3.3)	3.4 (1.8-12.3)	3.0 (1.2-45.0)	5.0 (4.8-9.9)	3.0 (1.9-9.2)	0.193
N (%)						
< 1.3	-	-	1 (2.9)	-	-	
1.3 - 11.5	4 (100.0)	9 (90.0)	32 (94.1)	3 (100.0)	6 (100.0)	
> 11.5	-	1 (10.0)	1 (2.9)	-	-	

LH IU/L (Median, range)	1.8 (1.73-3)	4.4 (0.42-7.8)	3.5 (0.15-26)	6.9 (6.5-8.8)	4.2 (2.8-6.6)	0.826
N (%)						
< 0.5	-	1 (10.0)	1 (2.9)	-	-	
0.5-10.0	4 (100.0)	9 (90.0)	30 (88.2)	3 (100.0)	6 (100.0)	
> 10.0	-	-	3 (8.9)	-	-	

n - number of subjects; Rh – allergic rhinitis; A/Rh –allergic asthma with rhinitis; FSH – follicle stimulating hormone; LH – luteinizing hormone.

**Figure 1:**
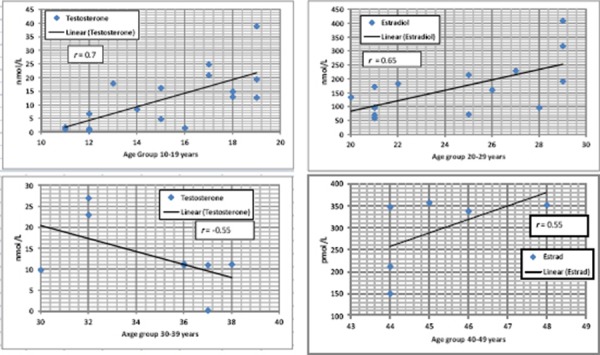
*Correlation between age and testosterone/estradiol in specific age group of patients*.

In patients divided into 4 groups according to age group, we found a strong positive correlation between age and testosterone only in the age group 10-19 years (Table 3), while moderate inverse correlation in the age group 30-39 years. Also a moderate correlation we found between age and estradiol in the age group 20-29 years and 40-49 years (Table 3) (Figure 1).

**Table 3:** Correlation between age and testosterone or estradiol in patients stratified according to their age in different age groups

Age group (years)	Testosterone (nmol/L)	Estradiol (pmol/L)
10-19	*r* = 0.7*	*r* = -0.23
20-29	*r* = -0.28	*r* = 0.65**
30-39	*r* = -0.55**	*r* = 0.11
49-49	*r* = -0.27	*r* = 0.55**
50-59	*r* = -1	*r* = 1

*r*- Pearsons correlation coefficient;

* strong;

** moderate.

Furthermore we did not find any correlation between testosterone/estradiol and having normal BMI being overweight or obese. However when the same variables were analyzed in the framework of specific disease group, we have found a strong positive correlation between age and testosterone/estradiol in the group of patients with asthma and weak to moderate correlation of age and estradiol levels in other allergic disease groups as well as controls (Table 4).

**Table 4:** Correlation between Age, BMI, testosterone and estradiol in male patients with different allergic diseases and healthy controls

Patients		Testosterone	Estradiol
A	Age	*r* = 0.77*	*r* = 0.99*

	BMI	*r* = - 0.3***	*r* = 0.35***

A/Rh	Age	*r* = 0.23	*r* =-0.24

	BMI	*r* = 0.16	*r* = -0.16

Rh	Age	*r*= -0.12	*r*= 0.48***

	BMI	*r* = - 0.33***	*r* = 0.27

D/U	Age	*r* = 0.17	*r* = 0.81*

	BMI	*r* = -0.57**	*r* = 0.16

C	Age	*r* = -0.05	*r* = 0.64**

	BMI	*r* = 0.42**	*r* = 0.32***

A-asthma; *r*- Pearsons correlation coefficient; A/Rh- asthma with rhinitis;

* strong; Rh- rhinitis;

** moderate; D/U- dermatitis or urticaria;

*** weak; C- controls.

**Figure 2:**
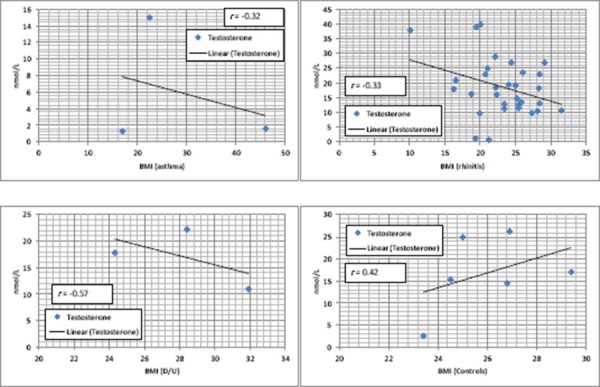
*Correlation between BMI and testosterone in patients and controls*.

BMI correlated weakly and inversely with testosterone in patients with asthma and rhinitis, moderately in patients with D/U and positively in control group (Figure 2). Eventually weak to no correlation was found between BMI and estradiol in each group of patients with particular allergic disease.

## Discussion

According to some studies, the accumulation of intra-abdominal fat and development of central obesity could be predicted by low serum total testosterone and vise versa [16-18]. On the other hand, more fat the human body has the more aromatase and consequently the more testosterone conversion to estradiol, hence the more estrogen [12]. Further, Testosterone levels are affected by age, body mass index (BMI) and co morbidities, such as type 2 diabetes and probably allergies [19]. Taking all these in consideration, we would expect that our obese allergic male patients, regardless of age, could have low levels of testosterone but high levels of estradiol.

However, the results obtained for each hormone within each research group and control group, were not consistent with our expectations. Thus the low average value of testosterone in the group of patients with A/Rh may be due to the high BMI, since 50% of them are overweight. And on the other hand, the low level of testosterone is associated in 30% of patients with the A/Rh with high levels of estradiol. Therefore, in this group of patients with the average age 28.2 years and BMI indicator for overweight, low levels of testosterone may be due to its conversion to estradiol in adipose tissue. Moreover, the concentrations of FSH and LH, which are analyzed to diagnose primary or secondary hypogonadism [20], were within normal range in 90% of patients with A/Rh, but the median value was 3.4 (range 1.8-12.3). Based on data from other studies, low testosterone and low to normal FSH and LH Levels may be indicative of Secondary hypogonadism [21, 22]. The group of patients with asthma, being significantly younger (mean 14.8 ± SD 2.8 year) with median BMI 22.4 (range 16.9-46), had lower levels of testosterone, FSH and LH while the highest level of estradiol, compared with other groups of patients and control group. Given that their average age fits in early to mid-puberty, when the concentration of testosterone may be from 1.04 to 3.47 nmol/L and 3.47 to 10.41 nmol/L, respectively [30-32], the concentration of testosterone in our asthmatics (Median 1.6 nmol/L range 1.3-15 nmol/L) was at the lower limit of the normal range. Despite the fact that the difference between groups was not statistically significant, low testosterone concentration and high concentration of estradiol in the asthma group was not due to high BMI, but may be the result of Secondary hypogonadism [21, 22]. Although we have not analyzed our patients any further for causes of Secondary hypogonadism, we can speculate that the reason may be the high level of cytochrome P450 aromatase in the adult male reproductive system or lack of receptors for estrogen (ERα) in the Leydig cells, based on numerous studies [23-27].

On the other hand, patients with Derm/Urticaria being in 66.6% of cases overweight and older (mean ± SD 11.8 34 years), were expected to have lower concentrations of testosterone and higher concentrations of estradiol, but on the contrary, the concentration of testosterone and estradiol were within normal limits, while FSH and LH concentration was higher than in other groups including the control group.

Moreover, in patients with allergic rhinitis being within the normal range for BMI and mean age similar to healthy control group, all hormones tested were within normal values.

Overall, in patients with A and A/Rh, low concentration of testosterone may be the cause of allergic symptoms, considering its immunsupresor activity reported in numerous researches [10, 28]. But the normal level of testosterone in allergic rhinitis and Derm/Urtic, can be explained by the fact that mast cells from different tissues may respond differently to the same biological factors [29].

So we can conclude that the lower the level of testosterone in men with allergy of respiratory system, the greater predisposition to clinical manifestation of symptoms of lower respiratory routes, while the higher level of testosterone, the greater the opportunity for localization only in the upper respiratory ways. The advantage of this research is that assigning hormone concentration is done in patients with allergies still not treated with glucocorticoids. Shortcoming of the research is the small number of participants within certain groups of allergic disease.

In conclusion, high BMI is not always associated with a low level of testosterone and high levels of estradiol in our patients with allergic diseases, but low levels of testosterone are present in patients with asthma and asthma/rhinitis although not among patients with rhinitis only. Our results should be confirmed in a larger group of participants.

## References

[1] Weinmayr G, Forastiere F, Büchele G, Jaensch A, Strachan DP, Nagel G (2014). Overweight/obesity and respiratory and allergic disease in children: international study of asthma and allergies in childhood (ISAAC) phase two. PLoS One.

[2] Mitchell EA, Beasley R, Björkstén B, Crane J, García-Marcos L, Keil U, ISAAC Phase Three Study Group (2013). The association between BMI, vigorous physical activity and television viewing and the risk of symptoms of asthma, rhinoconjunctivitis and eczema in children and adolescents: ISAAC Phase Three. Clin Exp Allergy.

[3] Schatz M, Zeiger RS, Zhang F, Chen W, Yang S-J, Camargo CA (2013). Overweight/obesity and risk of seasonal asthma exacerbations. J Allergy Clin Immunol Pract.

[4] Chen W, Mempel M, Schober W, Behrendt H, Ring J (2008). Gender difference, sex hormones, and immediate type hypersensitivity reactions. Allergy.

[5] Fantuzzi G (2005). Tissue, adipokines, and inflammation. J Allergy Clin Immunol.

[6] Kornizky Y, Topilsky M, Fireman E, Kivity S, Kivity S (1999). Specific IgE antibodies to aeroallergens and food among Israelis. Ann Allergy Asthma Immunol.

[7] Bouman A, Heineman MJ, Faas MM (2005). Sex hormones and the immune response in humans. Human Reproduction Update.

[8] Bebo BF, Schuster JC, Vandenbark AA, Offner H (1999). Androgens alter the cytokine profile and reduce encephalitogenicity of myelin-reactive T cells. J Immunol.

[9] Susan Kovats (2012). Estrogen receptors regulate an inflammatory pathway of dendritic cell differentiation: mechanisms and implications for immunity. Horm Behav.

[10] Osman M (2003). Therapeutic implications of sex differences in asthma and atopy. Arch Dis Child.

[11] Wang Ch, Jackson G, Jones TH, Matsumoto AM, Nehra A, Perelman MA (2011). Low Testosterone Associated With Obesity and the Metabolic Syndrome Contributes to Sexual Dysfunction and Cardiovascular Disease Risk in Men With Type 2 Diabetes. Diabetes Care.

[12] Mogri M, Dhindsa S, Quattrin T, Ghanim H, Dandona P (2013). Testosterone concentrations in young pubertal and post-pubertal obese males. Clinical Endocrinology.

[13] Boulet LP (2013). Asthma and obesity. Clinical & Experimental Allergy.

[14] Inseon S Choi (2011). Gender-Specific Asthma Treatment. Allergy Asthma Immunol Res.

[15] Vieira VJ, Ronan AM, Windt MR, Tagliaferro AR (2005). Elevated atopy in healthy obese women. Am J Clin Nutr.

[16] Allan CA, McLachlan RI (2010). Androgens and obesity. Curr Opin Endocrinol Diabetes Obes.

[17] MacDonald AA, Herbison GP, Showell M, Farquhar CM (2010). The impact of body mass index on semen parameters and reproductive hormones in human males: a systematic review with meta-analysis. Hum Reprod Update.

[18] Brand JS, van der Tweel I, Grobbee DE, Emmelot-Vonk MH, van der Schouw YT (2011). Testosterone, sex hormone-binding globulin and the metabolic syndrome: a systematic review and meta-analysis of observational studies. Int J Epidemiol.

[19] Yoo S, Kim HB, Lee SY, Kim BS, Kim JH, Yu JH, Kim BJ, Hong SJ (2010). Association between obesity and the prevalence of allergic diseases, atopy, and bronchial hyperresponsiveness in Korean adolescents. Int Arch Allergy Immunol.

[20] Christina Carnegie (2004). Diagnosis of Hypogonadism: Clinical Assessments and Laboratory Tests. Rev Urol.

[21] Griffin JE, Wilson JD (2001). Endocrinology & Metabolism. Disorders of the testes. Harrison’s principles of internal medicine.

[22] Beers MH, Berkow R (1999). The Merck Manual of Diagnosis and Therapy.

[23] Carreau S, Lambard S, Delalande C, Denis-Galeraud I, Bilinska B, Bourguiba S (2003). Aromatase expression and role of estrogens in male gonad: a review. Reprod Biol Endocrinol.

[24] Rago V, Bilinska B, Palma A, Ando S, Carpino A (2003). Evidence of aromatase localization in cytoplasmic droplet of human immature ejaculated spermatozoa. Folia Histochem Cytobiol.

[25] Lambard S, Galeraud-Denis I, Bouraima H, Bourguiba S, Chocat A, Carreau S (2003). Expression of aromatase in human ejaculated spermatozoa: a putative marker of motility. Mol Hum Reprod.

[26] Turner KJ, Macpherson S, Millar MR, McNeilly AS, Williams K, Cranfield M (2002). 2002. Development and validation of a new monoclonal antibody to mammalian aromatase. J Endocrinol.

[27] Vaucher L, Funaro MG, Mehta A, Singh SR (2014). Activation of GPER-1 Estradiol Receptor Downregulates Production of Testosterone in Isolated Rat Leydig Cells and Adult Human Testis. PLoS ONE.

[28] Canguven O, Albayrak S (2011). Do low testosterone levels contribute to the pathogenesis of asthma?. Med Hypotheses.

[29] Mu-oz-Cruz S, Mendoza-Rodríguez Y, Nava-Castro KE, Yepez-Mulia L, Morales-Montor J (2015). Gender-Related Effects of Sex Steroids on Histamine Release and FcεRI Expression in Rat Peritoneal Mast Cells. Journal of Immunology Research.

[30] Severson Alexia Testosterone Levels by Age. (2013, February 6).

[31] Vermeulen A, Kaufman JM (2002). Diagnosis of hypogonadism in the aging male. The Aging Male.

[32] Sacchetti M, Lambiase A, Moretti C, Mantelli F, Bonini S (2015). Sex Hormones in Allergic Conjunctivitis: Altered Levels of Circulating Androgens and Estrogens in Children and Adolescents with Vernal Keratoconjunctivitis. Journal of Immunology Research.

